# Multivariate meta-analysis of individual participant data helped externally validate the performance and implementation of a prediction model

**DOI:** 10.1016/j.jclinepi.2015.05.009

**Published:** 2016-01

**Authors:** Kym I.E. Snell, Harry Hua, Thomas P.A. Debray, Joie Ensor, Maxime P. Look, Karel G.M. Moons, Richard D. Riley

**Affiliations:** a Public Health, Epidemiology and Biostatistics, School of Health and Population Sciences, Public Health Building, University of Birmingham, Edgbaston, Birmingham B15 2TT, UK; b School of Mathematics, Watson Building, University of Birmingham, Edgbaston, Birmingham B15 2TT, UK; c Julius Center for Health Sciences and Primary Care, University Medical Center Utrecht, Str. 6.131, PO Box 85500, 3508 GA Utrecht, The Netherlands; d Dutch Cochrane Centre, University Medical Center Utrecht, Str. 6.131, PO Box 85500, 3508 GA Utrecht, The Netherlands; e Research Institute for Primary Care and Health Sciences, Keele University, Staffordshire ST5 5BG, UK; f Department of Medical Oncology, Erasmus MC Cancer Institute, Erasmus University Medical Center, PO Box 2040, 3000 CA Rotterdam, The Netherlands

**Keywords:** Risk prediction, Prognostic model, Individual participant data (IPD), Multivariate meta-analysis, External validation, Calibration, Discrimination, Heterogeneity, Model comparison

## Abstract

**Objectives:**

Our aim was to improve meta-analysis methods for summarizing a prediction model's performance when individual participant data are available from multiple studies for external validation.

**Study Design and Setting:**

We suggest multivariate meta-analysis for jointly synthesizing calibration and discrimination performance, while accounting for their correlation. The approach estimates a prediction model's average performance, the heterogeneity in performance across populations, and the probability of “good” performance in new populations. This allows different implementation strategies (e.g., recalibration) to be compared. Application is made to a diagnostic model for deep vein thrombosis (DVT) and a prognostic model for breast cancer mortality.

**Results:**

In both examples, multivariate meta-analysis reveals that calibration performance is excellent on average but highly heterogeneous across populations unless the model's intercept (baseline hazard) is recalibrated. For the cancer model, the probability of “good” performance (defined by C statistic ≥0.7 and calibration slope between 0.9 and 1.1) in a new population was 0.67 with recalibration but 0.22 without recalibration. For the DVT model, even with recalibration, there was only a 0.03 probability of “good” performance.

**Conclusion:**

Multivariate meta-analysis can be used to externally validate a prediction model's calibration and discrimination performance across multiple populations and to evaluate different implementation strategies.

**What is new?**

**Key findings**
• Given individual participant data (IPD) from multiple external validation studies, meta-analysis enables researchers to summarize prediction model performance, in terms of both average performance and consistency in performance across populations. It thereby allows different implementation strategies (e.g., recalibration) to be formally compared.
• A multivariate meta-analysis approach should be used to jointly evaluate discrimination and calibration performance, while accounting for their correlation. This can be used within internal–external cross-validation (to also incorporate a model development phase) or when IPD from multiple studies are available for external validation of existing models.

**What this adds to what was known?**
• Before implementation, risk prediction models require validation in data external to that used for model development. This is best achieved using IPD from multiple studies, so that model performance can be examined and quantified across multiple populations of interest. A good prediction model will have satisfactory performance on average across all external validation data sets and crucially little or no between-study heterogeneity in performance.
• Our examples show that a prediction model may have excellent average performance but with heterogeneity (inconsistency) in performance across populations. Recalibration of the model's intercept term (or baseline hazard) in the intended population might reduce heterogeneity and thereby improve the probability of acceptable model performance when applied in new populations.

**What is the implication and what should change now?**
• When IPD are available from multiple studies for external validation of a prediction model, researchers should use multivariate meta-analysis to jointly summarize calibration and discrimination performance and to identify how best to implement the model in new populations.

## 1 Introduction

A crucial part of medical research is to develop risk prediction models. These aim to accurately predict disease and outcome risk in individuals [1], [2], [3], thereby informing clinical diagnosis and prognosis. For example, healthy individuals with a high predicted risk of future disease (e.g., cardiovascular events) may be advised to modify their lifestyle and behavior choices (e.g., smoking, exercise), and diseased individuals may be grouped (e.g., stage of cancer) according to future outcome risk so that clinical decisions (such as treatment options, monitoring strategies) can be tailored accordingly. Two well-known examples are QRISK [4] and the Nottingham Prognostic Index [5]. They are typically implemented within a multivariable regression framework, such as logistic or Cox regression, which provides an equation to estimate an individual's risk based on values of multiple predictors (prognostic factors [6]) such as age, biomarkers, and genetic information.

A key stage of prediction model research is model development [2]. This identifies important predictors and develops the risk prediction equation using an available data set; it usually also examines the model's apparent performance in this same data or uses internal validation techniques (such as bootstrap resampling) to examine and adjust for optimism in performance [7]. The next stage is external validation [8], [9], [10]. This uses data external to the model development data and its source and examines whether the model predictions are accurate in another (but related) situation. The aim was to ascertain the model's generalizability to the intended populations for use [11] and to identify the best implementation strategy (e.g., recalibration of the intercept).

Unfortunately, most prediction research focuses on model development, and there are relatively few external validation studies [12]. However, nowadays, there is increasing access to multiple data sets, as evident in meta-analyses using individual participant data (IPD) from multiple studies [13], [14]. This provides an exciting opportunity to perform external validation on multiple occasions [15], [16]. Model development and external validation can even occur simultaneously, using an approach called internal–external cross-validation [17], [18]. This develops a model in all but one of the IPD studies, and then, its external validity is immediately checked in the omitted study. This process is repeated across all rotations of the omitted study, to measure external validity in each distinct IPD study.

Given multiple external validation studies, meta-analysis methods are needed to synthesize and summarize model performance appropriately across the available populations. Van Klaveren et al. [16], Pennells et al. [15], and, within internal–external cross-validation, Royston et al. [17] consider approaches to summarize validation performance across multiple studies or clusters. These focus mainly on producing pooled estimates of discrimination performance; that is, a model's ability to distinguish correctly between patients with and without the outcome of interest. Researchers should also be interested in summarizing calibration performance, which is the agreement between a model's predicted risk and the observed risk. Calibration is often ignored in external validation research [19], although it is fundamental that observed and predicted risks should closely agree. Moreover, baseline risk may vary across study populations, and so, a model's implementation may need to be tailored to each population (often referred to as recalibration) to improve calibration performance in new populations.

In this article, we propose multivariate meta-analysis for jointly synthesizing discrimination and calibration performance, while accounting for their correlation. This can be used within internal–external cross-validation (to also incorporate a model development phase) or when IPD from multiple studies are available for external validation of existing models. We show that the multivariate approach summarizes a prediction model's average discrimination and calibration performance and quantifies the heterogeneity in performance across populations. It also allows researchers to predict the potential calibration and discrimination of a model when it is applied to a new population and can be used to estimate the probability of “good” performance (as predefined by the user). Using two real examples, we illustrate how this enables researchers to compare the performance of different implementation strategies (e.g., recalibration of the intercept term) to help identify the best strategy for applying the model in practice.

The article now proceeds by introducing the proposed multivariate meta-analysis methodology for summarizing and comparing validation performance (Section 2). Two clinical examples are then used to illustrate the approach (Section 3), one for diagnosis and one for prognosis, and we conclude with some discussion (Section 4).

## 2 Meta-analysis of predictive performance statistics from multiple external validation studies

External validation of a prediction model requires evaluation of its predictive performance, in terms of both calibration and discrimination. There are many statistical measures available for this purpose [1], [20]. Here, we focus on those most commonly used: the C statistic [20], [21], the D statistic [22], [23], the calibration slope [1], [20], calibration-in-the-large [1], and the expected/observed number of events. These are defined in the Appendix at www.jclinepi.com. We focus here on how to meta-analyze such performance statistics when they are estimated in multiple external validation studies.

### 2.1 Obtaining suitable data for meta-analysis

The meta-analysis approach requires an estimate of each performance statistic of interest (e.g., C statistic, calibration slope) from each external validation study. Given IPD, these can be calculated in each validation study using appropriate statistical methods, as described elsewhere [1], [20], [23]. However, meta-analysis also requires the variance–covariance matrix of the performance statistics in each study: in other words, the variance of each performance estimate and (for multivariate meta-analysis) the correlation between all pairs of estimates. A general approach to obtain these is via nonparametric bootstrapping, as described in the Appendix at www.jclinepi.com.

### 2.2 Univariate random-effects meta-analysis

For clarity, before proposing our multivariate approach, we first describe a univariate random-effects meta-analysis that is applicable separately to each performance measure of interest [24], [25]. In external validation study *i*, let $Y_{ij}$ be the estimate of the *j*th performance statistic of interest, and let $\;S_{ij}^{2}$ be its sample variance (derived from bootstrapping and assumed known), then the univariate meta-analysis can be written as:

(1) $$\begin{array}{l} Y_{ij}\;∼\;N\left(\mu_{ij},\;S_{ij}^{2}\right)\\ \mu_{ij}\;∼\;N\left(\mu_{j},\tau_{j}^{2}\;\right) \end{array}$$

Equation (1) assumes the $Y_{ij}$ are normally distributed about the *i*th study's true validation performance, $\mu_{ij}$, and that the $\mu_{ij}$ are also normally distributed with an average of $\mu_{j}$ and a between-study standard deviation of $\tau_{j}$. There are several frequentist methods that can be used for estimation of a random-effects meta-analysis; here, we use restricted maximum likelihood (REML) [26]. With the addition of prior distributions for unknown parameters, a Bayesian approach is also possible, for example, using Gibbs sampling. An approximate 100(1-*α*)% confidence interval (CI) for the average performance, $\mu_{j}$, is obtained by ${\hat{\mu }}_{j}\;\pm 1.96\;SE\left({\hat{\mu }}_{j}\right)$, where SE$\left({\hat{\mu }}_{j}\right)$ is the standard error of ${\hat{\mu }}_{j}\text{.}$ White [27] proposed that $\text{SE}\left({\hat{\mu }}_{j}\right)$ is inflated to account for the uncertainty in the estimated $\tau_{j}$, and we implement this here.

### 2.3 Summarizing consistency in model performance

On its own, ${\hat{\mu }}_{j}$ is an incomplete summary because it does not adequately summarize the consistency in performance across studies. Estimates such as $I^{2}$ (the percentage of the total variation in study estimates that is due to between-study heterogeneity [28]) and ${\hat{\tau }}_{j}^{2}\;$ are thus also helpful [29]. However, when evaluating performance statistics of a risk prediction model, we are examining its generalizability, in other words, its robustness when applied in new populations that differ from those it was developed in [11]. Thus, consistency is best expressed by a 100(1−α)% prediction interval for the performance of the model in a new population [24], [25]. This is derived by

(2) $${\hat{\mu }}_{j}\;\pm \;\;t_{\propto ,\;N-2}\sqrt{{\hat{\tau }}_{j}^{2}\;+V\left({\hat{\mu }}_{j}\right)\;}$$

where $t_{\propto ,\;N-2}$ is the 100(1−∝/2 )% percentile of the *t*-distribution for *N* − 2 degrees of freedom (*N* = no. of studies), *V*$\left({\hat{\mu }}_{j}\right)$ = SE${\left({\hat{\mu }}_{j}\right)}^{2}$, and ∝ is typically taken to be 0.05 to give a 95% interval. The use of a *t*-distribution, rather than a normal distribution, is used to account for the uncertainty in ${\hat{\tau }}_{j}^{2}\;$ [24]. The prediction interval thus indicates the performance expected in a new (external validation) study, similar to those included in the meta-analysis.

### 2.4 Multivariate meta-analysis

Our multivariate approach is an extension of Equation (1) [30] and allows the joint synthesis of all predictive performance measures of interest from the *i* = 1 to *N* external validation studies, while accounting for their within- and between-study correlation. Let there be *j* = 1 to *J* measures of interest and let $Y_{i}$ be a vector containing the available *J* estimates $\left(Y_{i1},Y_{i2},\ldots ,Y_{iJ}\right)$ of the measures in the *i*th validation study. The general multivariate meta-analysis model is as follows:

(3) $$\begin{array}{l} Y_{i}\;\text{|}\theta_{i}\;∼\;\text{MVN}\left(\theta_{i},S_{i}\right)\\ \theta_{i}\;∼\;\text{MVN}\left(\mu ,\Sigma \right) \end{array}$$

Here, MVN denotes a multivariate normal distribution, $\theta_{i}$ contains the true underlying effects for the *J* performance measures for the *i*th study, $S_{i}$ is the within-study variance–covariance matrix for the *i*th study (assumed known) containing the *J* variances of the estimates (in the diagonal: $S_{i1}^{2},S_{i2}^{2},\ldots ,S_{iJ}^{2}$) and their covariances (in the off diagonal; e.g., $\rho_{Wi\left(1,2\right)}S_{i1}S_{i2}$ is the within-study covariance for measures 1 and 2, where $\rho_{Wi\left(1,2\right)}$ is their within-study correlation caused by estimates derived from the same patients), μ contains the *J* means for the measures of interest, and Σ is the between-study variance–covariance matrix containing the *J* between-study variances (in the diagonal$\tau_{1}^{2},\tau_{2}^{2},\ldots ,\tau_{J}^{2}$) and their between-study covariances (in the off diagonal; e.g., the between-study covariance for measures 1 and 2 is $\rho_{B\left(1,2\right)}\tau_{1}\tau_{2}$, where $\rho_{B\left(1,2\right)}$ is their between-study correlation induced by differences in study populations and settings). The number of rows in each vector is equal to the number of measures. In its simplest form with two measures of interest (e.g., C statistic and calibration slope), Equation (3) can be expressed as a bivariate meta-analysis (Appendix at www.jclinepi.com).

REML can again be used for estimation, although other options are available [30], [31]. Multivariate extensions to $I^{2}$ can also be calculated [26], [31], giving the fraction of the total variability due to between-study variability for each performance statistic ($I_{J}^{2}$).

### 2.5 Making joint inferences across multiple performance measures

After Equation (3) is estimated, marginal confidence and prediction intervals for each performance measure can be obtained using the formulae given in the univariate section. However, by accounting for their correlation, the multivariate approach also enables joint inferences. For instance, extending Equation (2) to a bivariate *t*-distribution with *k* – 2 degrees of freedom, one can obtain a joint 95% prediction region for two performance measures of interest (e.g., the C statistic and the calibration slope) in a new population. Joint probabilistic inferences can also be made if we assume the multivariate *t*-distribution is an approximate posterior distribution (i.e., we assume it is obtained from a Bayesian analysis with uninformative priors and give it means, variances, and covariances obtained from REML estimation of Equation (3)—see Supplementary Material 1/Appendix B at www.jclinepi.com for full details [32]). For example, one can derive the joint probability that the C statistic will be above 0.7 and the calibration slope will be between 0.9 and 1.1 in a new population. A fully Bayesian approach can also be used to derive such posterior inferences by formally specifying prior distributions and combining them with the likelihood, then using, for example, Gibbs sampling to take samples from the exact posterior distributions. Riley et al. [33] describe the Bayesian approach to multivariate meta-analysis with IPD.

### 2.6 Comparing the predictive performance of different implementation strategies

When applying a prediction model to a new population, different implementation strategies might be used regarding the choice of model intercept (baseline hazard). This is illustrated in Section 3.1 and Section 3.2, and, for example, includes recalibration. Meta-analysis of performance statistics allows such implementation strategies to be formally compared. The aim is to identify an implementation strategy that, for each performance measure, has excellent performance on average (indicated by ${\hat{\mu }}_{j}$); small values of between-study heterogeneity (indicated by ${\hat{\tau }}_{j}$ and/or $I_{J}^{2}$); and a narrow prediction interval that suggests consistently good performance in new populations. Multivariate meta-analysis even allows the competing strategies to be ranked according to their overall performance: for example, according to the joint probability that, in a new population, the C statistic will be above 0.7 and the calibration slope will be between 0.9 and 1.1. The strategy with the largest probability will be ranked first.

### 2.7 Meta-regression and examining covariates

Meta-analysis Equation (3) can be extended to a multivariate meta-regression that includes study-level covariates to explain between-study heterogeneity, such as treatment policies, population characteristics (e.g., mean age), year of investigation, and length of follow-up. Competing implementation strategies can then be evaluated and compared for specific subgroups of studies (e.g. those done within the last few years, those with consistent treatment policies, and those with the same case-mix, and so forth). This may help to identify populations where model performance is satisfactory and others where it is inadequate, to inform the model's generalizability and applicability [11]. A nice example of a meta-regression to examine the impact of case-mix variation on model performance is given by Pennells et al. [15], who identify that studies with a higher standard deviation of age are strongly associated with a higher C statistic and D statistic. Model performance can also be examined for patient-level covariates; for example, discrimination and calibration could be estimated for males and females separately. Equation (3) can then be applied to summarize each subgroup or even the difference between subgroups.

## 3 Applied examples

We now illustrate the proposed meta-analysis methods with two applied prediction model examples, one for diagnosis and one for prognosis, and compare the performance of different implementation strategies, including recalibration.

### 3.1 Diagnostic example: prediction of existing deep vein thrombosis (DVT)

#### 3.1.1 Data, model development, and competing implementation strategies

We used IPD from 12 studies to develop a diagnostic prediction model for the risk of having DVT in patients who were suspected of having DVT, as described previously [34]. A total of 10,002 patients were available across the 12 studies (with study sample sizes ranging from 153 to 1,768 patients), and 1,864 (19%) patients truly had DVT. This IPD is used here only for illustration purposes and not to develop or recommend the optimal diagnostic model to be used in medical practice.

The prediction model was developed using logistic regression, including a separate intercept for each study and three predictors chosen a priori: sex (male = 1, female = 0), surgery (recent surgery or bedridden = 1, no recent surgery or bedridden = 0), and calf difference (≥3 cm = 1, <3 cm = 0). However, three different implementation strategies were considered (for the model intercept) when applying the developed model to the external validation data set:

Strategy (1): Use a new intercept estimated in the external validation data set itself. This is a form of model recalibration [35].

Strategy (2): Use the estimated weighted average of the study intercept terms from the developed model.

Strategy (3): Use the estimated intercept for one of the studies in the developed model that had the most similar prevalence of DVT to the external validation study.

Internal–external cross-validation was undertaken for each implementation strategy, and their predictive performance then summarized and compared across the 12 external validation studies using our multivariate meta-analysis approach.

#### 3.1.2 Results

Regardless of which study was excluded, the predictor effect estimates (log odds ratios) were very similar in each cycle of the internal–external cross-validation approach [Supplementary Material 2/Appendix B at www.jclinepi.com shows the parameter estimates in each cycle, and the intercept to be implemented in strategy (3)]. During external validation of the model, for each implementation strategy, four validation statistics were estimated: calibration-in-the-large, calibration slope, the C statistic, and the ratio of expected and observed DVT cases, as defined in the Appendix at www.jclinepi.com. These results are shown (with standard errors) in Supplementary Material 3(A)/Appendix B at www.jclinepi.com for each of the strategies. Their within-study correlations, obtained from bootstrapping with 1,000 samples, are shown in Supplementary Material 3(B)/Appendix B at www.jclinepi.com. These are large (between +0.90 and +0.98) for the calibration slope and C statistic, indicating a strong positive relationship between them. In other words, as the observed calibration slope of model predictions decreases (becomes flatter), the observed discrimination of the model predictions also decreases (less separation); conversely, when model predictions produce a steeper observed calibration slope, the discrimination is improved. The other measures of calibration (calibration-in-the-large and expected/observed) measure overall agreement and thus are not affected so much by changes in discrimination; thus, their within-study correlation with the C statistic is close to zero. There is a perfect negative correlation between log(expected/observed) and calibration-in-the-large by definition.

**Fig. 1:**
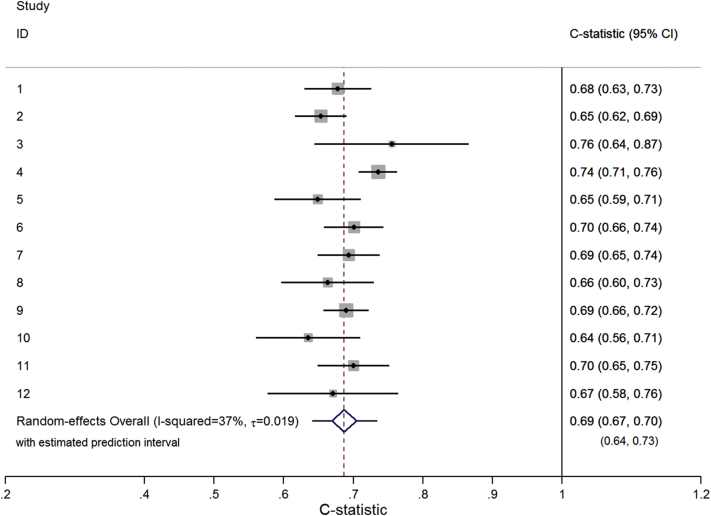
Forest plot showing the C statistic results from the trivariate random-effects meta-analysis result (Table 1) for the DVT prediction model implemented using strategy (2).

**Fig. 2:**
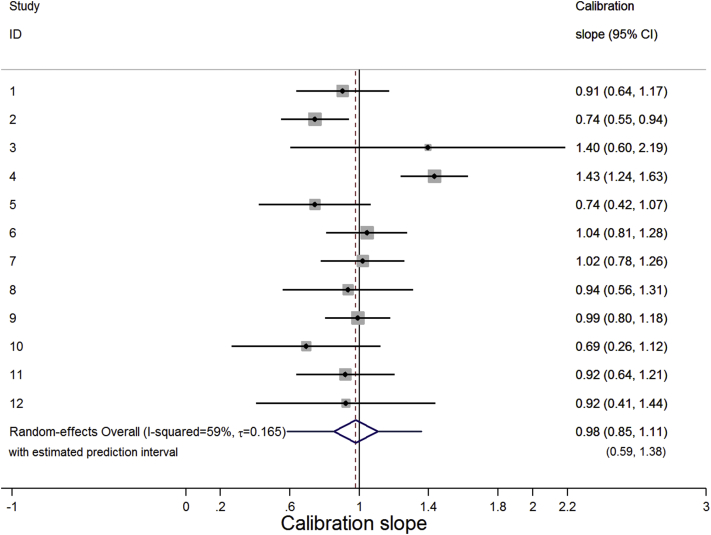
Forest plot showing the calibration slope result from the trivariate random-effects meta-analysis (Table 1) for the DVT prediction model implemented using strategy (2).

The multivariate meta-analysis results for each statistic are shown in Table 1. The meta-analysis results for the C statistic are practically the same in all implementation strategies, as are those for the calibration slope. The mean C statistic is 0.69 (95% CI: 0.67, 0.71), indicating moderate discrimination. There is a small amount of between-study heterogeneity ($\hat{\tau }$≈0.02; *I*^2^ ≈ 37%), leading to a 95% prediction interval of 0.64–0.73, revealing fairly consistent discrimination performance across studies (Fig. 1). The mean calibration slope is around 0.98 (95% CI: 0.85, 1.10), which is close to the ideal value of one although indicating very slight overprediction. The amount of between-study heterogeneity is large ($\hat{\tau }$≈0.16; *I*^2^ ≈ 59%), leading to a wide 95% prediction interval [e.g., 0.59–1.38 for strategy (2)]. This contains values well above and well below one, which, respectively, suggest that in some populations, the predicted probabilities vary too little (i.e., the model is underfitted and/or assigns probabilities that are too similar across individuals) and in others they vary too much (i.e., the model is overfitted to the development sample and assigns probabilities that vary too much across individuals). This illustrates how the average performance is an incomplete picture; calibration slope is good on average but could be poor in particular populations (Fig. 2).

Calibration-in-the-large does differ more importantly across implementation strategies (Table 1), as it is sensitive to the choice of intercept. The meta-analysis results reveal it is, on average, slightly worse for strategy (1) as there is a small overprediction in the proportion with DVT (−0.13; 95% CI: −0.19, −0.08). However, there is almost no heterogeneity in the calibration-in-the-large ($\hat{\tau }$=0.008; *I*^2^ = 1%), leading to a narrow 95% prediction interval (−0.20 to −0.07). Using strategy (2) or (3) the average calibration-in-the-large is closer to zero(−0.004 and 0.047, respectively) but comes at the expense of slightly larger between-study heterogeneity ($\hat{\tau }$=0.53 and 0.27, *I*^2^ = 97% and 89%, respectively), leading to wider prediction intervals. For example, for strategy (2), the 95% prediction interval is −1.24 to 1.23.

Instead of calibration-in-the-large, it is perhaps easier to interpret the expected/observed proportion of DVT cases (Table 1). This follows a similar pattern (Table 1), with narrowest prediction interval for strategy (1) and slightly improved average performance for strategies (2) and (3). The 95% prediction interval for expected/observed for strategy (1) suggests the overall agreement is likely to be reasonable in new populations (1.05–1.14), with the number of DVT cases overpredicted by between 5% and 14%. However, the 95% prediction interval is unsatisfactory for the other strategies; for example, it is 0.41–2.54 for strategy (2) indicating the number of predicted DVT cases in a new population could range from 59% too few up to 154% too many.

Overall, therefore, strategy (1) appears best as it removes heterogeneity in the calibration-in-the-large and expected/observed, and maintains similar discrimination across populations. However, the prediction model would benefit from additional predictors, as current discrimination is only moderate and there is large heterogeneity in calibration slope. This is confirmed by a joint probability of only 0.03 that strategy (1) will give a C statistic ≥0.7 and a calibration slope between 0.9 and 1.1 in a new population (Table 2). If the criteria for model discrimination is relaxed to a C statistic ≥0.65, then the joint probability improves but only to 0.43.

### 3.2 Prognostic example: prediction of mortality in breast cancer patients

#### 3.2.1 Data, model development, and competing implementation strategies

We used IPD from eight cohort studies (relating to eight different countries from Look et al. [36]) to develop and evaluate a prognostic prediction model for the risk of mortality over time in women recently diagnosed with breast cancer. In total, there were 7,435 patients (ranging from 69 patients to 3,242 per study) and 2,043 events. The maximum follow-up duration was 120 months, and the median follow-up duration across all studies was 86.3 months. Internal–external cross-validation was used, and, in each cycle, a Royston–Parmar flexible parametric survival model was fitted [37], [38], [39], with the baseline cumulative hazard function modeled using restricted cubic splines (with four knots deemed sufficient) and predictor effects (hazard ratios) assumed constant over time. A set of eight candidate predictors was considered at each cycle: age, tumor type, tumor grade, tumor size, number of positive nodes, menopausal status, adjuvant therapy, and hormone receptor status. Backward selection was used, with *P* > 0.05 taken for exclusion. Separate but proportional baseline hazard functions were included for each country; that is, one study was taken as the reference group, and others were allowed a country-specific adjustment factor. When applying the developed model to the external validation study, three different implementation strategies were considered (in regard the baseline hazard):

Strategy (1): Use a new country-specific adjustment factor as estimated in the validation study itself. This is a form of recalibration but assumes the baseline hazard in the validation study and the development studies are proportional.

Strategy (2): Use a weighted average of the estimated country-specific adjustment factors from the developed model.

Strategy (3): Use the country-specific adjustment factor for a country that was included in the developed model and is closest geographically to the validation country.

Internal–external cross-validation was undertaken for each strategy, and their predictive performance then summarized and compared across the eight external validation studies using meta-analysis.

#### 3.2.2 Results

The predictor effect estimates (log hazard ratios) were similar in each cycle of the internal–external cross-validation approach (results available on request). The backward selection retained all candidate predictors in each cycle, apart from menopausal status that was always excluded. For each implementation strategy, we evaluated model performance in each external validation study by estimating Harrell's C statistic [20], the D statistic [22], [40], and the calibration slope between the predicted hazard function and the observed hazard function, as defined in the Appendix at www.jclinepi.com. The estimates, with their variances and within-study correlation, are shown in Supplementary Material 4/Appendix B at www.jclinepi.com. Within-study correlations were all positive and generally moderate to large.

Multivariate meta-analysis of the validation statistics is summarized in Table 3 for each implementation strategy. The summary C statistic and D statistic results are barely affected by the choice of strategy. The average C statistic is 0.71, and its 95% prediction interval is 0.66–0.76, suggesting consistently moderate discrimination across populations. The average D statistic is about 0.33, which equates to a moderate hazard ratio of 1.39 (95% CI: 1.23, 1.57) between two equal sized groups across the prognostic index. However, D is inconsistent across populations (*I*^2^ is about 87%), and thus, its prediction interval is wide (Table 3).

Calibration slope is affected by the choice of strategy. For strategy (1), which allows recalibration in the validation study, the calibration slope is excellent. The meta-analysis gives an average calibration slope of 1.003, with only moderate heterogeneity (*I*^2^ = 35%) leading to a narrow prediction interval of 0.93–1.08. In contrast, strategies (2) and (3) perform poorly. Although average calibration is excellent, there is large between-study heterogeneity [e.g., *I*^2^ = 99% for strategy (3)] leading to wide predictions intervals [e.g., 0.15–1.77 for strategy (3)]. This again reveals how average performance is an incomplete and potentially misleading summary of performance.

**Fig. 3:**
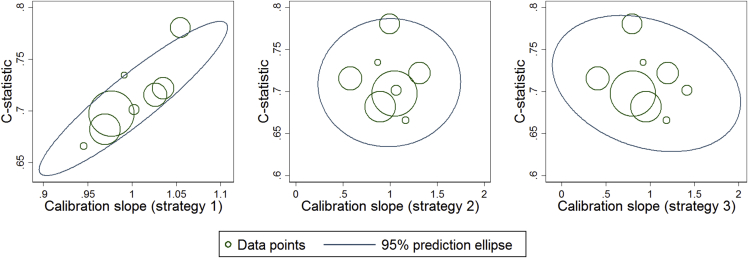
Summary of validation performance of the breast cancer model for each implementation strategy, with regard to the C statistic and the calibration slope results from the trivariate meta-analysis (Table 3).

Fig. 3 shows joint prediction ellipses for the C statistic and the calibration slope, derived using the multivariate meta-analysis results for each strategy. For implementation strategy (1), there is a joint probability of 0.67 for a C statistic ≥ 0.7 and a calibration slope between 0.9 and 1.1; however, the probability is only 0.15 for strategy (3) and 0.22 for strategy (2).

Strategy (1) thus performs best, but it requires recalibration of the model in new countries and may be difficult to implement. We therefore sought to improve strategy (2), which does not include recalibration, by identifying the cause of heterogeneity in its calibration performance. It was observed that study 3 gave the poorest calibration slope on external validation of the models, most likely due to the baseline hazard in study 3 being different in shape (nonproportional) to those other studies. Extending Equation (3) to a multivariate meta-regression with a covariate for country (1 = study 3, 0 = otherwise) explained a large part of the heterogeneity (*P* < 0.001). We repeated the internal–external cross-validation approach for strategy (2) but omitted study 3 for the entire process. External validation performance was improved, as heterogeneity in calibration slope was reduced ($\hat{\tau }$ = 0.156 excluding study 3, $\hat{\tau }$ = 0.22 including study 3), and thus, its 95% prediction interval was narrower (Supplementary Material 5/Appendix B at www.jclinepi.com). The joint probability for a C statistic ≥0.7 and a calibration slope between 0.9 and 1.1 was improved to 0.32 but still considerably worse than strategy (1), indicating recalibration remains preferable.

## 4 Discussion

We have proposed a multivariate meta-analysis approach for summarizing and comparing prediction model performance across multiple external validation studies using IPD. This can be used within internal–external cross-validation to also incorporate a model development phase or when IPD from multiple studies are available for external validation of existing models. Each of the statistical methods involved (such as obtaining within-study correlations and fitting the multivariate equation) only take up to a few minutes to perform using computer software such as Stata (Texas, USA) and provide results that improve the interrogation of a prediction model's performance and its implementation strategy.

Currently, most external validation research is undertaken using a single data set. However, multivariate meta-analysis of IPD is a novel way to examine the overall performance and generalizability of a prediction model across multiple data sets [13], [15], [16]. A good model will have satisfactory performance on average across all external validation data sets. But ideally, there should also be little or no between-study heterogeneity in performance. Our examples showed that a prediction model may have excellent average performance but may not have consistent performance across data sets. Such heterogeneity is rarely considered in external validation research but should be routinely examined where possible, in particular to identify the best implementation strategy. In our examples, the investigation of heterogeneity revealed that recalibration of the intercept term to the validation population was essential; otherwise, there was considerable inconsistency in calibration performance of our prediction models. The importance of intercept recalibration is also shown elsewhere [41], [42]. However, it may not entirely remove the issue of miscalibration, as seen in the DVT example where there remained slight overprediction even after recalibration. In particular, if there is also heterogeneity in predictor effects, then one may also need to recalibrate these to the intended population; however, this defeats the purpose of the initial research (i.e., to develop a prediction model that can be used widely and easily) and rather indicates that additional and/or more homogenous predictors are required.

Heterogeneity in discrimination performance was also observed in our examples. This may also be due to heterogeneity in predictor effects across populations and/or different case-mix distributions across populations, as populations with wider ranges of continuous predictors often have better discrimination [15]. For such reasons, incorporating matched case–control studies alongside cohort studies may increase heterogeneity in discrimination performance, as the former typically have narrower ranges of predictors [43]. Another potential cause of heterogeneity in performance of a prognostic prediction model is follow-up time, and also, heavy censoring may bias Harrell's C statistic, prompting Gönen and Heller [44] to propose an alternative. Such factors may also impact the magnitude of between-study correlation in the performance measures.

As external validation of a prediction model usually requires multiple statistical measures of performance, in particular at least one for calibration and one for discrimination [8], [19], our multivariate meta-analysis approach jointly synthesizes all measures together across multiple validation studies. This accounts for their within-study and between-study correlation [45], which may arise because measures are highly related [33]. For example, the C statistic and D statistic typically have moderate to large positive within-study correlation [as seen in Supplementary Material 4(B)/Appendix B at www.jclinepi.com] as they are both measures of discrimination and within studies are estimated on the same patients. Similarly, the calibration slope and C statistic may also be correlated between studies, for instance, if the between-study heterogeneity in predictor effects causes calibration slope to become greater than 1 as discrimination improves but less than 1 as discrimination worsens. Accounting for such correlation in the meta-analysis allows the borrowing of strength across performance measures to potentially reduce bias and improve precision [46], [47]. Furthermore, it is crucial to account for correlation when computing joint probabilities of model performance, such as the magnitude of the C statistic and calibration slope, as otherwise inferences may be misleading [45].

Our intention was to illustrate how the multivariate meta-analysis approach allows researchers to summarize both discrimination and calibration. We focused on well-known statistical criteria, such as the calibration slope, (Harrell's) C statistic, and Royston and Sauerbrei's D statistic. However, we recognize that the criteria for a “good” prediction model is open to much debate [48], and readers may prefer to meta-analyze other statistical measures available, including alternatives to Harrell's C statistic [44]. Clinical criteria may also be preferred [49], to focus more on the consequences for decision making [50]. Whatever criteria are used, we recommend they are prespecified in a published protocol [51]. Visual plots of calibration [23] and discrimination [52] are also important, as neatly illustrated by Royston et al. [17]. Calibration estimates can also be obtained (and then meta-analyzed) for particular subgroups within studies, for example, defined by particular patient characteristics or categories of the prognostic index [23]. Also, we note that excellent validation performance is not the end of the story: a prediction model's impact on patient outcomes also needs to be evaluated, for example, in subsequent trials [3].

A potential limitation of our work is the multivariate normality assumption for the distribution of true performance across studies. Although this is a common assumption in the meta-analysis field, prediction intervals and regions are potentially vulnerable to departures from this [53]. A related issue is the choice of scale to use for the estimates of validation performance [16], and further research is needed on this. Internal–external cross-validation is also limited if the number of studies are small, and researchers should ensure the number of events is suitable in each cycle [54], [55].

In conclusion, we propose multivariate meta-analysis for external validation of the performance and implementation of a prediction model when IPD are available for multiple studies. The approach encourages researchers to focus not only on average performance, but also on the consistency in performance across populations, for both calibration and discrimination.

## Figures and Tables

**Table 1:** Trivariate meta-analysis results^a^ for the calibration and discrimination performance of the DVT model for each implementation strategy

Strategy	Validation statistic	Estimate (95% CI) of mean, μ	95% Prediction interval	*I*^2^ (%)	$\hat{\tau }$ (95% CI)
Strategy (1): Develop using logistic regression and implement with intercept estimated in external validation study	Calibration-in-the-large	−0.130 (−0.185, −0.075)	−0.195, −0.065	1	0.008
Calibration slope	0.975 (0.855, 1.097)	0.597, 1.353	57	0.158
Log(expected/observed)	0.086 (0.047, 0.124)	0.041, 0.128	0	0.0009
C statistic	0.687 (0.670, 0.704)	0.645, 0.729	34	0.017
Strategy (2): Develop using logistic regression and implement with average study intercept taken from developed model	Calibration-in-the-large	−0.004 (−0.313, 0.305)	−1.240, 1.232	97	0.532
Calibration slope	0.980 (0.853, 1.107)	0.585, 1.375	59	0.165
Log(expected/observed)	0.022 (−0.206, 0.250)	−0.887, 0.931	97	0.391
C statistic	0.687 (0.669, 0.705)	0.640, 0.734	37	0.019
Strategy (3): Develop using logistic regression and implement with intercept taken from a study used in development data with a similar prevalence	Calibration-in-the-large	0.047 (−0.120, 0.214)	−0.584, 0.678	89	0.270
Calibration slope	0.976 (0.851, 1.102)	0.578, 1.375	59	0.167
Log(expected/observed)	−0.029 (−0.150, 0.093)	−0.485, 0.427	89	0.195
C statistic	0.687 (0.669, 0.705)	0.640, 0.734	38	0.019

*Abbreviations*: DVT, deep vein thrombosis; CI, confidence interval.

a A trivariate meta-analysis was fitted to calibration-in-the-large, calibration slope, and C statistic and then again for log(expected/observed), calibration slope, and C statistic. Perfect negative correlation between calibration-in-the-large and expected/observed within studies prevents all four measures being analyzed together (due to collinearity). Results were practically the same for calibration slope and C statistic, regardless of the trivariate model fitted.

**Table 2:** Joint predicted probability of “good” discrimination and calibration performance of the DVT model for each of the three implementation strategies, derived using the multivariate meta-analysis results for the C statistic and calibration slope shown in Table 1

Calibration slope required	Minimum C statistic required	Joint predicted probability of meeting criteria in new population		
Strategy (1): Develop using logistic regression and implement with intercept estimated in external validation study	Strategy (2): Develop using logistic regression and implement with average study intercept taken from developed model	Strategy (3): Develop using logistic regression and implement with intercept taken from a study used in development data with a similar prevalence
0.9–1.1	0.70	0.027	0.037	0.037
0.8–1.2	0.70	0.146	0.158	0.156
0.9–1.1	0.65	0.427	0.413	0.409
0.8–1.2	0.65	0.728	0.712	0.707

*Abbreviation*: DVT, deep vein thrombosis.

**Table 3:** Trivariate random-effects meta-analysis results for calibration and discrimination performance of the breast cancer model for each implementation strategy

Strategy	Validation statistic	Pooled estimate (95% CI)	95% Prediction interval	*I* squared (%)	Estimate of *τ*	Joint probability of “good”^a^ performance in a new population
Strategy (1): Develop using Royston–Parmar and implement with baseline hazard estimated in validation study	Calibration slope	1.003 (0.971, 1.036)	0.927, 1.080	35	0.026	0.67
C statistic	0.711 (0.690, 0.733)	0.657, 0.766	49	0.019
D statistic	0.328 (0.215, 0.442)	−0.056, 0.713	87	0.146
Strategy (2): Develop using Royston–Parmar model and implement with the estimated average baseline hazard from developed model	Calibration slope	0.994 (0.835, 1.153)	0.411, 1.577	98	0.224	0.22
C statistic	0.711 (0.691, 0.732)	0.662, 0.761	43	0.017
D statistic	0.332 (0.212, 0.452)	−0.080, 0.745	88	0.157
Strategy (3): Develop using Royston–Parmar model and implement with the estimated baseline hazard from the closest geographical country	Calibration slope	0.961 (0.741, 1.181)	0.148, 1.775	99	0.313	0.15
C statistic	0.710 (0.687, 0.734)	0.653, 0.767	50	0.020
D statistic	0.330 (0.211, 0.450)	−0.068, 0.728	87	0.151

*Abbreviation*: CI, confidence interval.

a Defined by a C statistic ≥0.7 and an calibration slope between 0.9 and 1.1.
