# Supplementary material for: Multivariate meta-analysis of individual participant data helped externally validate the performance and implementation of a prediction model
Source: J Clin Epidemiol. 2016 Jan;69:40–50. doi: 10.1016/j.jclinepi.2015.05.009 (PMC4688112; doi:10.1016/j.jclinepi.2015.05.009)
Supplement: Appendix A [file mmc1.docx]

**Appendix**: Brief explanation of key statistical concepts considered in the article

**C-statistic**

A measure of a prediction model's discrimination (separation) between those with and without an event (outcome), which can be calculated for either binary or survival outcome data [[20](#_ENREF_20), [21](#_ENREF_21)]. Also known as the concordance index or, for binary outcomes, the area under the receiver operating characteristic (ROC) curve. It gives the probability that for any randomly selected pair of individuals, one with and one without the event (outcome), the model assigns a higher probability to the individual with the event (outcome). A value of 1 indicates the model has perfect discrimination, whilst a value of 0.5 indicates the model discriminates no better than chance. For the DVT model we used the ‘roctab’ module in STATA to calculate the C-statistic, whilst for the breast cancer model we used ‘stcstat2’ to calculate Harrell’s C-statistic.

**D-statistic**

A measure of discrimination for time-to-event outcomes [[22](#_ENREF_22)]. This can be interpreted as the log hazard ratio comparing two equally sized groups defined by dichotomising at the median value of the prognostic index from the developed model (where the prognostic index is defined by the combined predictor effects in the developed model, i.e. beta1*X1 + beta2*X2 + ...). Higher values for the D-statistic indicate greater discrimination, and an increase of 0.1 over other risk scores is suggested to be a good indicator of improved prognostic separation [[10](#_ENREF_10)]. For the breast cancer model, the D-statistic in the external validation study was calculated using the ‘str2d’ module [[40](#_ENREF_40)] in STATA after fitting the Royston-Parmar model in our development data using the ‘stpm2’ module [[38](#_ENREF_38)].

**Calibration slope**

This is a measure of agreement between observed and predicted risk of the event (outcome) across the whole range of predicted values [[1](#_ENREF_1), [20](#_ENREF_20)]. For example, if a prediction model is developed using logistic regression it is of the form:

logit(p) = alpha + beta1*X1 + beta2*X2 + ...

Then the predicted probability (P-pred) is derived using

P-pred = exp(alpha + beta1*X1 + beta2*X2 + ...) / [1 + exp(alpha + beta1*X1 + beta2*X2 + ...)]

In the validation data, fitting the model

logit(p) = delta0 + delta1*(logit(P-pred))

is used to calculate the calibration slope (delta1), which should ideally be 1 (though a value of 1 does not by itself confirm calibration is perfect [[48](#_ENREF_48)]).

In the breast cancer example in our article, we used a flexible parametric survival model to develop the prediction model, using the Royston-Parmar approach where the log cumulative hazard function is modelled using restricted cubic splines [[37-39](#_ENREF_37)], with four knots chosen. To estimate calibration slope of the developed model, we fitted the following model in each external validation study:

lnH(t) = gamma0+gamma1*z1+gamma2*z2+gamma3*z3 +b*X

where b is the calibration slope, lnH(t) is the log cumulative hazard function over time, t, and the gamma and z terms define the knots and the baseline lnH(t). The (gamma1*z1+gamma2*z2+gamma3*z3) value was forced to be that from the developed model; the X value for each patient corresponds to their value of the prognostic index from the developed model (i.e. Beta1*X1 + Beta2*X2 + ...); and the gamma0 value is dependent on the implementation strategy used. In strategy (1) gamma0 is newly estimated in the external validation study (akin to recalibration). In strategy (2) gamma0 is forced to be a weighted (meta-analysis) average of all the study-specific gamma0 estimates from the developed model. In strategy (3) the gamma0 value is forced to be the gamma0 estimate for the nearest country available in the developed model.

**Calibration-in-the-large**

This is an overall measure of calibration,[[1](#_ENREF_1)] defined by fitting in the validation dataset

logit(p) = alpha0 + offset

where alpha0 is the calibration-in-the-large and the offset term is equal to logit(P-pred)

**Expected/Observed number of events (E/O)**

This is another measure of calibration, closely related to the calibration-in-the-large, but more intuitive to interpret. It provides the ratio of the total expected events (outcomes) to the total observed events (outcomes). It can be obtained by summing all predicted probabilities in the validation dataset and dividing by the number of observed events. An ideal value is 1. Values less than 1 indicate the model is under-predicting the total number of events in the population, whilst values above 1 indicate it is over-predicting the total events in the population.

**Non-parametric bootstrapping to obtain variance-covariance matrix of performance estimates**

Consider a single external validation study. Bootstrapping uses the IPD from this study and randomly selects one patient with replacement, then randomly selects a second patient with replacement, and repeats until the same sample size is obtained as in the original study. This process is repeated *b* times, so that *b* bootstrap samples are obtained. Then, in each of the bootstrap samples the performance statistics of the developed model are estimated (e.g. $\text{Y}_{\text{i}1}$ could be the estimated C-statistic and $\text{Y}_{\text{i}2}$ could be the estimated calibration slope estimate in study *i*). This produces *b* values for each performance statistic. The observed variance of the *b* values for each statistic estimates their variance (i.e. it gives $\text{ S}_{\text{i}1}^{2}$, the variance of $\text{Y}_{\text{i}1}$ , etc). Similarly, the observed correlation across the *b* samples for each pair of validation statistics estimates their within-study correlation (i.e. it gives $\rho_{Wi(\text{ }1,2)}$, the within-study correlation between $\text{Y}_{\text{i}1}$ and $\text{Y}_{\text{i}2}$ etc).

**Bivariate model**

When there are two performance measures of interest (e.g. C-statistic and calibration slope), the general multivariate meta-analysis of equation (3) can be simplified to the following bivariate meta-analysis:
