# Supplementary material for: Multivariate meta-analysis of individual participant data helped externally validate the performance and implementation of a prediction model
Source: J Clin Epidemiol. 2016 Jan;69:40–50. doi: 10.1016/j.jclinepi.2015.05.009 (PMC4688112; doi:10.1016/j.jclinepi.2015.05.009)
Supplement: Appendix B [file mmc2.doc]

Supplemental Material (Online Only)


Supplementary material 1: Example of SAS syntax to derive joint probabilities of model performance with regard to two performance measures.

The joint probability of 'good' performance in a new population is obtained below by calculating the proportion of 1000000 samples drawn from the approximate posterior bivariate t-distribution (with k - 2 d.f.; k = no. of studies). The parameters values to use are derived from the estimates of the mean vector and its variance-covariance matrix, and the
between-study variance-covariance matrix, following REML estimation of multivariate meta- analysis equation (3). SAS code is provided below based on using the 'RANDMVT' module . Alternatively this can be performed using the “mnormt” package in R software [32].

/* Breast cancer example: strategy (1)*/
proc iml;
load module=randmvt;
/* set random number seed */
call randseed(1);
/* define number of samples to take */ N=100000;
/* enter degrees of freedom (no. of studies minus 2)*/ DF = 6;
/* Define the means of the posterior distribution to be the meta-analysis mean estimates (mu) for the C-statistic and calibration slope*/
Mean = {0.711 1.003};
/* Define the variance-covariance matrix S for the posterior distribution by: V_11 = Variance for C-statistic = tau1-squared + var(mu1)
V_22 = Variance for calibration slope = tau2-squared + var(mu2)
V_12 = V_21 = Covariance of the C-statistic and calibration slope	= tau12 +
cov(mu1,mu2)
(where tau1 and tau2 are the between-study SD for the C-statistic and the calibration slope, respectively; mu1 and mu2 are the mean estimates of the C- statistic and the calibration slope, respectively; var(mu1) and var(mu2) are the variances of the estimates mu1 and mu2, respectively; tau12 is the between-study covariance; and cov(mu1,mu2) is the covariance of the estimates of mu1 and mu2) */ S = {0.00048 0.000611, 0.000611 0.000958};
/* derive the samples and define them by x*/
x = RANDMVT( N, DF, Mean, S );
/* show the 1000 pair of values generated if on interest*/
print x;

/* convert x to a dataset called samples */
create samples from x;
append from x;
quit;

/* Create a second dataset that identifies whether 'good' performance was obtained for each sample within x - here 'good' is defined by the C-statistic > 0.7 and the calibration slope between 0.9 and 1,1 */
data samples2;
set samples;
/* C-statistic criteria */
if col1 > 0.7 then y =1;
/* calibration criteria */ if col2 > 0.9 then z1 = 1; if col2 < 1.1 then z2 = 1;
/* joint criteria */
if y=1 and z1=1 and z2 =1 then accept=1;
else accept=0;
run;

/* Calculate the joint probability of good performance, simply by the mean value of the 'accept' variable - which is the proportion of samples that had the 'good' performance */
proc means;
vars accept;
run;


Supplementary  material 2: Model parameter estimates for the fitted DVT model to be implemented using strategy (3), which fits a logistic

regression model in each cycle of the internal-external cross-validation approach to obtain predictor effects and study-specific intercepts.


1)	(â2)
(â3 )


Note: Bold numbers represent the intercept used for external validation in the excluded study for strategy (3), where the intercept from the study with the closest prevalence was selected.


Supplementary material 3(a): Estimates (standard errors) of the calibration and discrimination performance of the DVT model developed in each cycle of the internal-external cross-validation approach, for the three implementation strategies


Study used for external validation	
Strategy (1):
Develop using logistic regression and apply with intercept estimated in external validation study

CITL 	Calibration
slope 	Log(E/O) 	C-statistic	Strategy (2):
Develop using logistic regression and apply with average study intercept taken from developed model
Calibration
CITL 	slope 	Log(E/O) 	C-statistic	Strategy (3):
Develop using logistic regression and apply with intercept taken from a study used in development data with a similar prevalence

CITL 	Calibration 	Log(E/O) 	C-statistic slope	

1

2

3

4

5

6

7

8

9

10

11

12	-0.172 	0.903 	0.140 	0.678
(0.098) 	(0.131) 	(0.080) 	(0.024)
-0.051 	 0.741 		0.028 	 0.653 (0.079) 	(0.100) 	(0.042) 	(0.019)
-0.172 	1.418 	0.135 	0.761
(0.224) 	(0.397) 	(0.174) 	(0.055)
-0.084 	 1.432 		0.060 	 0.735 (0.054) 	(0.100) 	(0.039) 	(0.014)
-0.185 	 0.742 		0.141 	 0.649 (0.122) 	(0.164) 	(0.094) 	(0.031)
-0.149 	 1.030 		0.112 	 0.699 (0.082) 	(0.114) 	(0.062) 	(0.021)
-0.178 	1.017 	0.156 	0.694
(0.090) 	(0.117) 	(0.080) 	(0.023)
-0.115 	 0.932 		0.081 	 0.663 (0.133) 	(0.189) 	(0.093) 	(0.035)
-0.139 	 0.994 		0.098 	 0.690 (0.068) 	(0.099) 	(0.048) 	(0.017)
-0.127 	 0.695 		0.103 	 0.636 (0.150) 	(0.215) 	(0.122) 	(0.037)
-0.135 	 0.921 		0.094 	 0.701 (0.111) 	(0.140) 	(0.078) 	(0.026)
-0.197 	0.936 	0.160 	0.673
(0.191) 	(0.269) 	(0.155) 	(0.048)	-0.440 	0.905 	0.349 	0.678
(0.098) 	(0.136) 	(0.081) 	(0.025)
1.105 	 0.745 	 -0.709 	  0.653 (0.078) 	(0.100) 	(0.043) 	(0.019)
-0.292 	1.396 	0.223 	0.756
(0.225) 	(0.405) 	(0.176) 	(0.057)
0.488 	 1.434 	 -0.367 	  0.736 (0.057) 	(0.099) 	(0.040) 	(0.014)
-0.267 	 0.744 		0.201 	  0.649 (0.125) 	(0.165) 	(0.096) 	(0.032)
-0.055 	 1.044 		0.042 	  0.701 (0.084) 	(0.119) 	(0.064) 	(0.022)
-0.877 	1.020 	0.732 	0.694
(0.089) 	(0.122) 	(0.078) 	(0.023)
0.464 	 0.936 	 -0.340 	  0.663 (0.129) 	(0.192) 	(0.090) 	(0.034)
0.118 	 0.991 	 -0.084 	  0.689 (0.073) 	(0.096) 	(0.052) 	(0.017)
-0.081 	 0.693 		0.066 	  0.635 (0.144) 	(0.219) 	(0.116) 	(0.038)
0.258 	 0.921 	 -0.185 	  0.700 (0.111) 	(0.145) 	(0.078) 	(0.026)
-0.570 	0.923 	0.440 	0.671
(0.190) 	(0.264) 	(0.155) 	(0.048)	0.114 	0.905 	-0.094 	0.678
(0.094) 	(0.132) 	(0.078) 	(0.024)
0.583 	 0.736 	 -0.344 	 0.652 (0.084) 	(0.102) 	(0.045) 	(0.019)
-0.042 	1.390 	0.036 	0.755
(0.223) 	(0.408) 	(0.173) 	(0.057)
0.031 	 1.434 	 -0.022 	 0.736 (0.057) 	(0.102) 	(0.040) 	(0.014)
0.016 	 0.749 	 -0.011 	 0.650 (0.121) 	(0.159) 	(0.093) 	(0.031)
0.187 	 1.035 	 -0.144 	 0.700 (0.084) 	(0.119) 	(0.063) 	(0.022)
-0.399 	1.014 	0.344 	0.693
(0.090) 	(0.123) 	(0.080) 	(0.024)
-0.038 	 0.943 		0.028 	 0.665 (0.132) 	(0.192) 	(0.092) 	(0.034)
-0.132 	 0.996 		0.093 	 0.690 (0.070) 	(0.104) 	(0.050) 	(0.017)
0.447 	 0.699 	 -0.373 	 0.637 (0.146) 	(0.208) 	(0.119) 	(0.036)
0.132 	 0.916 	 -0.093 	 0.700 (0.110) 	(0.140) 	(0.077) 	(0.026)
-0.458 	0.930 	0.359 	0.672
(0.193) 	(0.256) 	(0.157) 	(0.046)	
Note: CITL refers to calibration-in-the-large and log(E/O) refers to log of the Expected/Observed number of events.

Supplementary material 3(b): Within-study correlations (ñWi  ), obtained through bootstrapping, between performance statistics estimated for the DVT model in each cycle of
the internal-external cross-validation approach, for the three implementation strategies


Study
CITL & calibration slope

CITL &
log(E/O)

CITL & C-statistic
Calibration slope & log(E/O)
Calibration slope &
C-statistic

Log(E/O) & C-statistic


Strategy (1): Develop using logistic regression and apply with intercept estimated in external validation study


Strategy (2): Develop using logistic regression and apply with average study intercept taken from developed model


Strategy (3): Develop using logistic regression and apply with intercept taken from a study used in development data with a similar prevalence
1 	-0.006 	-1.000 	-0.022 	0.006 	0.961 	0.021
2 	-0.032 	-1.000 	-0.046 	0.033 	0.977 	0.046
3 	-0.001 	-0.999 	0.009 	0.001 	0.955 	-0.011
4 	0.118 	-1.000 	0.045 	-0.117 	0.919 	-0.045
5 	0.046 	-1.000 	0.029 	-0.046 	0.983 	-0.029
6 	-0.010 	-1.000 	-0.045 	0.011 	0.948 	0.043
7 	0.047 	-1.000 	0.002 	-0.047 	0.912 	-0.003
8 	0.071 	-1.000 	0.032 	-0.072 	0.953 	-0.034
9 	-0.005 	-1.000 	-0.011 	0.005 	0.980 	0.011
10 	0.108 	-1.000 	0.064 	-0.108 	0.900 	-0.064
11 	0.000 	-1.000 	-0.025 	0.002 	0.956 	0.026
12 	-0.035 	-1.000 	-0.029 	0.036 	0.980 	0.030
1 	-0.051 	-1.000 	-0.054 	0.053 	0.960 	0.054
2 	0.046 	-0.990 	0.037 	-0.054 	0.976 	-0.035
3 	-0.051 	-0.999 	-0.037 	0.052 	0.958 	0.037
4 	0.062 	-0.999 	-0.009 	-0.065 	0.928 	0.014
5 	0.069 	-1.000 	0.055 	-0.069 	0.981 	-0.056
6 	0.031 	-1.000 	-0.013 	-0.031 	0.959 	0.013
7 	0.018 	-0.999 	-0.025 	-0.013 	0.923 	0.025
8 	0.105 	-0.999 	0.042 	-0.106 	0.951 	-0.038
9 	0.074 	-1.000 	0.068 	-0.074 	0.976 	-0.068
10 	0.035 	-1.000 	0.012 	-0.034 	0.895 	-0.012
11 	0.053 	-1.000 	0.015 	-0.052 	0.953 	-0.012
12 	0.000 	-0.999 	-0.001 	-0.003 	0.981 	-0.003
1 	0.005 	-1.000 	-0.012 	-0.004 	0.963 	0.013
2 	-0.006 	-0.997 	-0.033 	0.002 	0.978 	0.034
3 	-0.053 	-0.999 	-0.049 	0.054 	0.959 	0.050
4 	0.082 	-1.000 	0.015 	-0.082 	0.925 	-0.014
5 	0.034 	-1.000 	0.022 	-0.033 	0.982 	-0.022
6 	0.063 	-1.000 	0.020 	-0.065 	0.956 	-0.019
7 	-0.014 	-1.000 	-0.025 	0.015 	0.925 	0.025
8 	-0.002 	-1.000 	-0.052 	0.001 	0.954 	0.051
9 	-0.010 	-1.000 	-0.021 	0.011 	0.981 	0.021
10 	0.020 	-0.999 	-0.043 	-0.024 	0.892 	0.043
11 	0.038 	-1.000 	-0.001 	-0.040 	0.956 	0.000
12 	-0.036 	-0.999 	-0.041 	0.036 	0.980 	0.039

Note: CITL refers to calibration-in-the-large and log(E/O) refers to log of the Expected/Observed number of events.


Supplementary  material 4(a): Estimates (standard errors) of calibration and discrimination performance for the breast cancer model in each

cycle of the internal-external cross-validation approach, for the three implementations strategies


Study excluded for external validation


C-statistic1


D-statistic1


Strategy (1): Develop using Royston- Parmar model and implement with baseline hazard estimated in validation study
Calibration slope2
Strategy (2):
Develop using Royston-Parmar  model and implement with the estimated average baseline hazard in develop model


Strategy (3): Develop using Royston- Parmar model and implement with the estimated baseline hazard from the closest geographical country


1	0.697 (0.008)	0.493 (0.027)	0.977 (0.012)	1.049 (0.012)	0.805 (0.012)	
2	0.701 (0.036)	0.420 (0.117)	1.002 (0.057)	1.066 (0.057)	1.414 (0.056)	
3	0.715 (0.023)	0.106 (0.056)	1.026 (0.036)	0.578 (0.037)	0.405 (0.037)	
4	0.735 (0.068)	0.326 (0.187)	0.991 (0.097)	0.870 (0.098)	0.919 (0.097)	
5	0.666 (0.050)	0.238 (0.168)	0.946 (0.088)	1.168 (0.086)	1.184 (0.086)	
6	0.682 (0.017)	0.182 (0.041)	0.969 (0.037)	0.896 (0.038)	0.951 (0.037)	
7	0.781 (0.027)	0.280 (0.063)	1.054 (0.052)	0.996 (0.053)	0.794 (0.054)	
8	0.722 (0.016)	0.541 (0.058)	1.035 (0.030)	1.315 (0.029)	1.197 (0.030)	

1 The C-statistic and D-statistic only depend on the prognostic index (see Appendix). As the prognostic index (beta terms) from the developed model is not dependant on the implementation strategy, the C-statistic and D-statistic estimates are identical regardless of the implementation strategy used.
2 obtained as defined in the Appendix.


Supplementary material 4(b): Within-study correlations (ñWi), obtained using bootstrapping, between performance statistics estimated for the breast cancer model in each internal-external cross-validation cycle, for each implementation strategy


Country


1
2
3
4
5
6
7
8
Strategy (1): Baseline hazard estimated in external validation dataset. Strategy (2): Average baseline hazard from developed model.
Strategy (3): Baseline hazard from country included in the development, closest in proximity.

Supplementary material 5: Trivariate random-effects meta-analysis results of calibration and discrimination performance for the breast cancer model excluding Study 3, for implementation
strategy (2)


Strategy	Validation statistic


Pooled estimate (95% CI)


95% prediction interval


I- squared


Estimate of ô
Joint probability of good*
performance in a new population


Strategy (2) including Study 3: Develop using

Calibration slope

0.994 (0.835 to 1.153)

0.711 (0.691

0.411 to
1.577

98%	0.224

Royston-Parmar
model and apply with the estimated average
C-statistic
to 0.732)	0.662 to 0.76	43%	0.017
0.22

baseline hazard from developed model
D-statistic	0.332 (0.212
to 0.452)
-0.08 to
0.744
88%	0.157


Strategy (2) excluding Study 3: Develop using

Calibration slope

0.999 (0.883 to 1.114)

0.712 (0.688

0.594 to
1.404

95%	0.146

Royston-Parmar model and apply with the estimated average
C-statistic
0.650 to
to 0.735)	0.773
52%	0.021

0.32

baseline hazard from developed model
D-statistic	0.372 (0.256
to 0.490)
-0.014 to
0.760
85%	0.138


* defined by a C-statistic≥0.7 and a calibration slope between 0.9 and 1.1
